# Characterizing differences in the muscle transcriptome between cattle with alternative *LCORL-NCAPG* haplotypes

**DOI:** 10.1186/s12864-025-11665-z

**Published:** 2025-05-14

**Authors:** Fernanda Martins Rodrigues, Leif E. Majeres, Anna C. Dilger, Joshua C. McCann, Christopher J. Cassady, Dan W. Shike, Jonathan E. Beever

**Affiliations:** 1 Department of Animal Sciences, University of Illinois at Urbana-Champaign, Urbana, IL USA; 2 Present Address: Division of Biological and Biomedical Sciences, Washington University in Saint Louis, Saint Louis, MO USA; 3 Present Address: Department of Animal Science, Iowa State University, Ames, IA USA; 4 Department of Animal Science and Large Animal Clinical Sciences, University of Tennessee Institute of Agriculture, Knoxville, TN USA

**Keywords:** Beef, Bovine, Muscle, Fat, *LCORL*

## Abstract

**Background:**

The *LCORL-NCAPG* locus is a major quantitative trait locus (QTL) on bovine chromosome 6 (BTA6) that influences growth and carcass composition in cattle. To further understand the molecular mechanism responsible for the phenotypic changes associated with this locus, twenty-four Charolais-sired calves were selected for muscle transcriptome analysis based on alternative homozygous *LCORL-NCAPG* haplotypes (i.e., 12 “*QQ*” and 12 “*qq*”, where “*Q*” is a haplotype harboring variation associated with increased growth). At 300 days of age, a biopsy of the longissimus dorsi muscle was collected from each animal for RNA sequencing.

**Results:**

Gene expression analysis identified 733 genes as differentially expressed between *QQ* and *qq* animals (*q-*value < 0.05). Notably, *LCORL* and genes known to be important regulators of growth such as *IGF2* were upregulated in *QQ* individuals, while genes associated with adiposity such as *FASN* and *LEP* were downregulated, reflecting the increase in lean growth associated with this locus. Gene set enrichment analysis demonstrated *QQ* individuals had downregulation of pathways associated with adipogenesis, alongside upregulation of transcripts for cellular machinery essential for protein synthesis and energy metabolism, particularly ribosomal and mitochondrial components.

**Conclusions:**

The differences in the muscle transcriptome between *QQ* and *qq* animals imply that muscle hypertrophy may be metabolically favored over accumulation of fat in animals with the *QQ* haplotype. Our findings also suggest this haplotype could be linked to a difference in *LCORL* expression that potentially influences the downstream transcriptional effects observed, though further research will be needed to confirm the molecular mechanisms underlying the associated changes in phenotype.

**Supplementary Information:**

The online version contains supplementary material available at 10.1186/s12864-025-11665-z.

## Introduction

Quantitative trait loci (QTL) mapping in livestock animals allows the identification and characterization of genes underlying phenotypic variation in traits of economic relevance. In cattle, a QTL on bovine chromosome 6 (BTA6), the *LCORL-NCAPG* locus, has been identified by many independent studies across different cattle populations and breeds, as having a major influence on growth and composition. More specifically, this locus has been associated with body frame size [1–9], birth weight [2, 3, 7, 10–15], carcass weight [5, 10, 16–21], carcass composition [3, 5, 10, 17, 20, 22], and postnatal growth [11, 14, 15, 17, 22, 23]. There is a trend toward an increase in lean muscle and relative decrease in subcutaneous fat [3, 5, 17, 20], though marbling tends to be unaffected [3, 10, 17, 20], or is perhaps slightly reduced [5].

Setoguchi et al. [20] reported the association of this QTL with variation in carcass weight, ribeye area, and back fat, and refined its position to a 591-kb region that comprised four candidate genes: family with sequence similarity 184, member B (*FAM184)*; *DDB1* and *CUL4* associated factor 16 (*DCAF16*); non-SMC condensin I complex subunit G (*NCAPG*); and ligand dependent nuclear receptor corepressor-like (*LCORL*). They also identified a polymorphism in the *NCAPG* gene, *NCAPG c.1326T > G*, that produced a nonsynonymous amino acid substitution (*Ile442Met*). This polymorphism was proposed by several studies to be the putative quantitative trait nucleotide (QTN) causing the phenotypic variation observed [13, 20, 22].

However, other research suggests the QTN responsible is yet to be determined. Gutiérrez-Gil et al. [2] attempted to refine the position of the QTL, which they had previously associated with birth weight, birth body length, and bone weight [3]. Of the four sires identified as heterozygous for the QTL in their study, only two possessed a heterozygous genotype for the *NCAPG c.1326T > G* polymorphism, while two were homozygous for the *c.1326G* allele.

In our previous work, a genome wide association study (GWAS) of growth- and carcass-related phenotypes using 1,645 Simmental-Angus steers observed significant associations with the haplotype encompassing the *LCORL-NCAPG* locus and the phenotypes under investigation [24]. Due to the extensive linkage disequilibrium (LD) surrounding this locus and the fact that most of the significant haplotypes were found closer to the 3’ end of *NCAPG*, it was necessary to confirm whether the putative QTN was in phase with the haplotype in this population. To this end, the 82 sires were genotyped for the *NCAPG c.1326T > G* polymorphism. In this study, *Q* was defined as the haplotype causing the significant effect on the traits studied, and *q* as the ancestral haplotype(s). All sires expected to have at least one copy of the *Q* haplotype had at least one copy of the *c.1326G* allele. However, although the majority of the *qq* sires were homozygous for the *c.1326T* allele, five of them were heterozygous, revealing the presence of the *c.1326G* allele among *q* haplotypes. Additionally, three *Qq* sires were homozygous for the *c.1326G* allele. This supports the conclusions of Gutierrez-Gil et al. [3] that the *NCAPG* polymorphism is not the causative mutation underlying this QTL. Instead, we suggest that another polymorphism is responsible for these effects [24].

The *LCORL-NCAPG* locus has also been found to be influential on lean growth and body size in several other species, including humans [25–34], horses [35–39], dogs [40, 41], pigs [42], chickens [43, 44], goats [45, 46], and sheep [47, 48]. The fact that the orthologs of this locus are similarly implicated across species underscores its importance for growth and development in animals. A study in dogs revealed a loss of function mutation for the long isoform of *LCORL* exclusive to larger-sized breeds [40]. Other studies suggest this may also be the case in goats [45, 46], and a loss of function mutation associated with growth has been recently discovered in cattle as well [49–51].

This long isoform of *LCORL* has been characterized as an accessory protein for polycomb repressive complex 2 (PRC2) [52]. PRC2 is known to play a key role in establishing and maintaining cellular identity by silencing regions of the genome, including important genes for development of the body plan such as the *Hox* genes [53, 54]. Currently, no functional data exists on the activity of LCORL as an accessory protein to PRC2 [55], however its homology shared with its paralog encoded by *LCOR* suggests that it may be able to allosterically activate PRC2 and increase repressive activity [52].

While the evidence for a loss of function of the long *LCORL* isoform makes a compelling explanation for the increase in growth associated with this locus, there also is reason to believe that differences in the expression of the shorter isoform of *LCORL* may be involved as well. An ancestral retrocopy insertion event has resulted in equids having an additional 17 to 35 extra copies of the short isoform of *LCORL* that account for most *LCORL* expression in horses [56]. Furthermore, it was estimated the retrotransposition of these copies took place 18 million years ago, coinciding with dramatic increases in size and skeletal changes in equids. This evidence implies a potential link between an increase in expression as the retrocopies went through duplication events and these anatomical changes.

In cattle, Khansefid et al. [57] found that SNPs within 50 kb of *LCORL* were significant not only for changes in growth, but also were cis-eQTL, affecting the level of expression of the gene. While the LD surrounding this locus in cattle makes identification of a causative mutation extremely difficult, there are several variants upstream of *LCORL* that are associated with the increased growth haplotype. It is possible that some of these could be regulatory, and the change in *LCORL* expression may drive the change in phenotype.

Genetic variation at the *LCORL-NCAPG* locus causes permanent stable alterations in the developmental program of individuals throughout life. We hypothesize that the phenotypes associated with this QTL have unique molecular signatures that can be detected by transcriptional variation. While the specific QTN responsible for the phenotype associated with this locus and how it mediates its effects are unknown at this time, further characterization of the transcriptional differences associated with changes at this locus may provide insight into the mechanism underlying how this locus is able to regulate animal growth. Therefore, we used RNA-seq to characterize the transcriptional differences between individuals with alternative haplotypes at the *LCORL-NCAPG* locus.

## Materials and methods

### Selection of animals

All cattle used in this study were sourced Dixon Springs Agricultural Center owned by the University of Illinois. All procedures conducted were in accordance with protocols approved by the Institutional Animal Care and Use Committee of the University of Illinois (IACUC Protocol #17292 and #19118). Though the specific QTN remains presently unknown, there is a strong selection signature surrounding it, and it is known to segregate at high frequencies in certain breeds. In our previous work [58], we characterized the haplotype associated with increased lean growth (*Q*), and its defining variants that were not present in other animals that had other haplotypes segregating in this region (collectively, *q*) in 34 Charolais-sired cattle. The *Q* haplotype as it will be used in this paper will refer to the 814-kb haplotype containing the 217 variants exclusive to this haplotype, as well as all other variants they are in LD with. The haplotypes considered *qq* are any haplotypes that do not have these defining variants. Among the defining variants is rs384548488, the frameshift variant causing a predicted loss-of-function to the long isoform of *LCORL*. To characterize the differences in the muscle transcriptome between animals of the selected-upon haplotype in comparison with their contemporaries, 24 calves were selected based on their haplotype in this region. Of these, 12 were homozygous *QQ* and 12 homozygous *qq*, with both sexes equally represented in each haplotype group. Phenotype data was collected from a contemporary group of 344 Charolais-sired calves, including the 24 selected for the muscle biopsy used for the RNA-seq experiment. These calves were genotyped using on the Illumina^®^ BovSNP50 BeadChip (50K).

### Sample collection and RNA isolation

At 300 days of age, a biopsy sample of the longissimus dorsi muscle was collected from each of the 24 selected calves. All muscle biopsies conducted for this study were performed with lidocaine anesthetic in accordance with the IACUC protocols associated with this study. Between 100 and 200 mg of muscle were taken from the left side, 5 cm cranial to the hook bone and halfway between the axis and transverse processes of the lumbar vertebrae using a biopsy needle (Bard MAGNUM; 12-gauge x 16 cm). Tissue samples were transferred directly to cryotubes and snap frozen in liquid nitrogen.

Total RNA was extracted from the muscle samples using TRIzol^®^ Reagent (Life Technologies, Carlsbad, CA) according to the manufacturer’s protocol. Then, 5 µg of total RNA was purified using the RNeasy Mini Kit (Qiagen, Inc., Valencia, CA). Sample quality was assessed using an Agilent Bioanalyzer 2100 (Agilent Technologies, Santa Clara, CA). High quality samples (RIN > 7.0) were used to construct sequencing libraries.

### Library Preparation and RNA-Sequencing

Twenty-four Next Generation Sequencing (NGS) libraries were constructed using the Illumina’s TruSeq Stranded mRNA Library Preparation Kit (Illumina, Inc., San Diego, CA). All 24 libraries were indexed, pooled, and quantitated by qPCR. The pool was sequenced over four lanes for 101 cycles on an Illumina^®^ HiSeq 2500 (single-end chemistry) using a HiSeq SBS sequencing kit, version 4. Fastq files were processed and demultiplexed with bcltofastq 1.8.4 (Illumina, CA).

Adapter sequences and low-quality bases and reads were trimmed using Trimmomatic version 0.39 [59], with the following parameters: HEADCROP:1 ILLUMINACLIP:2:30:7 LEADING:24 TRAILING:24 SLIDINGWINDOW:10:28 MINLEN:50. Reads were assessed for quality with FastQC version 0.11.9 [60] and mapped to the ARS-UCD 2.0 assembly of the bovine genome using STAR version 2.7.6a [61], using the following non-default parameters: --seedSearchStartLmax 31 --outFilterScoreMinOverLread 0.5 --outFilterMatchNminOverLread 0.5. Read counts were obtained using the featureCounts function of the Rsubread package version 2.12.2 [62], using the default parameters. Multi-mapped reads, which are assigned a map quality score of 5 or lower by STAR, were removed using samtools [63]. This dataset is available under GEO accession number GSE98736.

### RNA-sequencing data analysis

Data analysis was conducted using R version 4.3.1 (R Core Team 2023) and packages as described below.

### Quality control and normalization

Quality control and normalization of the raw counts obtained from featureCounts was performed using edgeR version 3.42.4 [64]. Genes were filtered if they had fewer than 1.5 counts per million (CPM) in at least 12 samples, reducing the list of genes from 32,637 annotated genes to 11,783 genes that were considered for downstream analysis. Normalization factors were calculated using the Trimmed Mean of M-values (TMM) normalization method [65] in edgeR to account for compositional biases in libraries between each pair of samples.

### Statistical analysis of differential gene expression

An initial model was constructed using haplotype (*QQ* or *qq*) and sex. To estimate and correct for unknown batch effects, SVA version 3.48.0 was used [66]. Using the num.sv() function with the “be” method, a set seed of 04302023, the initial model, and normalized logCPM, SVA predicted 5 surrogate variables. Surrogate variables were then estimated using the sva() function, using the initial model and a null model that included only sex.

The five surrogate variables were used along with sex and haplotype for the final model for differential gene expression analysis in edgeR. Samples were fitted to a negative-binomial general linear model and tested for differential gene expression between the two haplotype groups using a likelihood-ratio test. The *p*-values of differential expression tests were corrected for multiple-hypothesis testing using Benjamini-Hochberg false discovery rate (FDR) correction. The threshold for significance was set to FDR *q*-value < 0.05.

### Gene set enrichment analysis (GSEA)

To render biologically meaningful insight from the expression differences observed between haplotype groups, gene set enrichment analysis (GSEA) was performed [67] using the clusterProfiler package (version 4.8.3) [68]. The input used was a ranked list of genes by their log-fold change in expression between haplotype categories. Mitochondrial genes not removed by filtering were manually renamed to their more conventional nomenclature (e.g. KEH36_p03 was renamed to ND5). After assigning genes to Entrez IDs using org.Bt.eg.db [69], 11,570 genes remained for further analysis, as the other 213 could not be assigned an ID. GSEA was carried out using gene ontology (GO) terms, as accessed from org.Bt.eg.db [69–71]. The seed set for every GSEA was 05172024. For these analyses, *p*-values were adjusted by Benjamini-Hochberg FDR correction, with the adjusted threshold of *p* < 0.05. Semantic similarity analysis using pairwise_termsim() from the enrichplot package version 1.20.3 [72] was employed to further simplify the GO terms to broader biological categories, using the default parameters.

### Statistical analysis of phenotype data

Growth and carcass phenotype data was collected from a contemporary group of 344 Charolais-sired calves, including the 24 used for the muscle RNA-seq experiment. Carcass phenotype data was collected at harvest, which took place at around 14–18 months of age. This phenotype data is available in Table S6 (Additional File 4). lmer() [73] was used to construct linear mixed-effects models to test for the association of the *Q* haplotype with phenotype in this population. As these calves were genotyped using the Bovine 50k BeadChip, Hapmap33628-BTC-041023 (rs110834363 / Chr6:37,505,093 T > C) was used as a surrogate for haplotype, as this variant was previously found to be exclusive to the *Q* haplotype [58], and is in close proximity and LD with the putative loss-of-function variant rs384548488 found on Chr6:37,401,770. Haplotype, alongside sex and days on feed were treated as fixed effects. Sire was used as a random effect to control for potential background genetic variance that could be contributing to phenotypic variation in addition to haplotype. Feeding pen was also included in the model as a random variable. The association between genotype for rs110834363 and 10 phenotypes was tested. Phenotypes included birth weight (BW), weaning weight (WW), yearling weight (YW), average daily gain (ADG), dry matter intake (DMI), hot carcass weight (HCW), ribeye area (REA), backfat thickness (BF), kidney pelvic heart fat % (KPH), and marbling score (MS).

## Results

### RNA-sequencing

Muscle biopsies from twenty-four Charolais-sired calves were selected for RNA-sequencing based on alternative haplotypes for the *LCORL-NCAPG* locus, with twelve *QQ* (homozygous for the selected-upon haplotype associated with increased lean growth) and twelve *qq* (both carried alleles are diverse ancestral haplotypes). Single-end sequencing yielded a total of 898,353,420 reads (average of 37.4 million reads per library). After removal of low-quality reads and adapter sequences, a total of 819,580,442 reads remained and were aligned to the ARS-UCD2.0 bovine reference genome. Final alignment rate after removal of multi-mapped reads was 95%, or an average of 32.8 million reads per sample. About 90.3% of these were assigned to a gene by featureCounts, resulting in an average of 29.6 million counts per animal for analysis.

### Removal of batch effects

SVA estimated five surrogate variables to remove batch effects that were subsequently used in the statistical model for further analyses. The SV value assigned to each sample can be viewed in Figure S1, Additional File 1. It appears that a possible driver for surrogate variable correction may be the unaccounted-for variation in tissue composition. For example, *LEP* and *FASN*, genes that are highly expressed in adipocytes, had a high degree of variability across samples, with individuals like 175A, 362A, and 238A having noticeably high expression of these genes when compared with their contemporaries (Figure S2, Additional File 2, panels A and B). These differences were substantial enough to make potentially high-fat samples outliers on the unadjusted principal component analysis (PCA) plot. SVA was able to detect and adjust for these outliers, while preserving the correlation between adiposity and sex, which was then removed separately. After adjusting for surrogate variables and sex, PC1 corresponded very closely to haplotype; this PC explained 19.25% of variance (Figure S2, Additional File 2, panel I). Notably, even after SV correction, sample 482A continued to cluster with *QQ*, despite being genotyped as *qq*.

### Differential gene expression

**Fig. 1:**
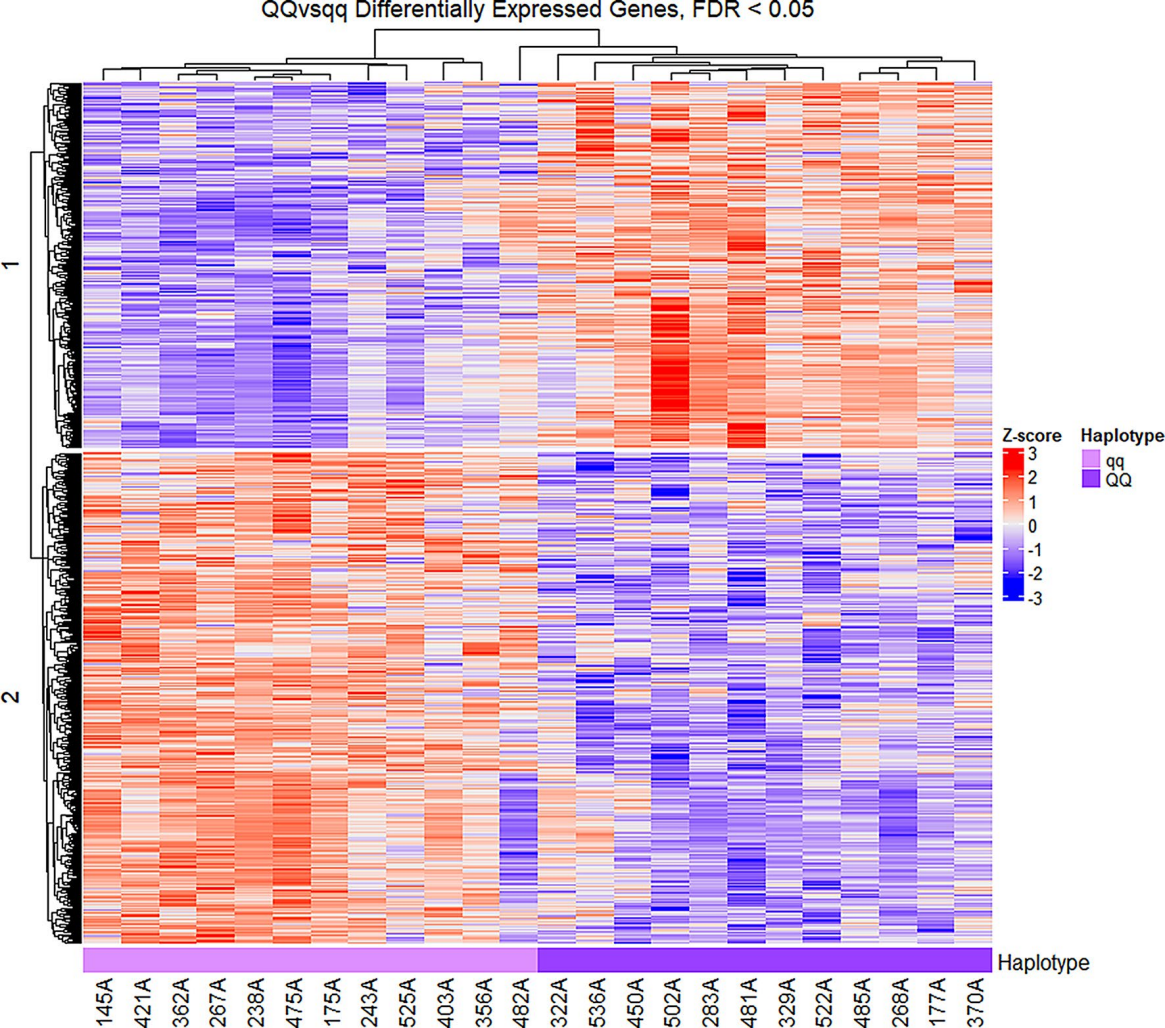
Heatmap of differentially expressed genes between *QQ* and *qq* animals. Scaled heatmap of the 733 differentially expressed genes identified in *QQ* vs. *qq* animals (*q*-value < 0.05). Each row represents one gene, and each column represents a sample. Z-transformed log2 of counts per million was used as the expression measure, with red denoting increased expression and blue indicating decreased expression relative to the mean for that gene in that sample. Samples of the same haplotype cluster together, with the exception of 482A.

In total, 733 genes were found to be differentially expressed between *QQ* and *qq* calves (*q*-value < 0.05). Of these genes, 420 of them were downregulated in *QQ*, and 313 were upregulated. A complete table with all likelihood-ratio test results including log-fold change and *q*-values for all genes is available in Table S1, Additional File 3. To visualize the per-sample levels of expression for the differentially expressed genes, a heatmap using the z-transformed logCPM was generated (Fig. 1). It can be observed here that sample 482A has a somewhat intermediate expression profile between *QQ* and *qq*.

### Gene set enrichment analysis (GSEA)

**Fig. 2:**
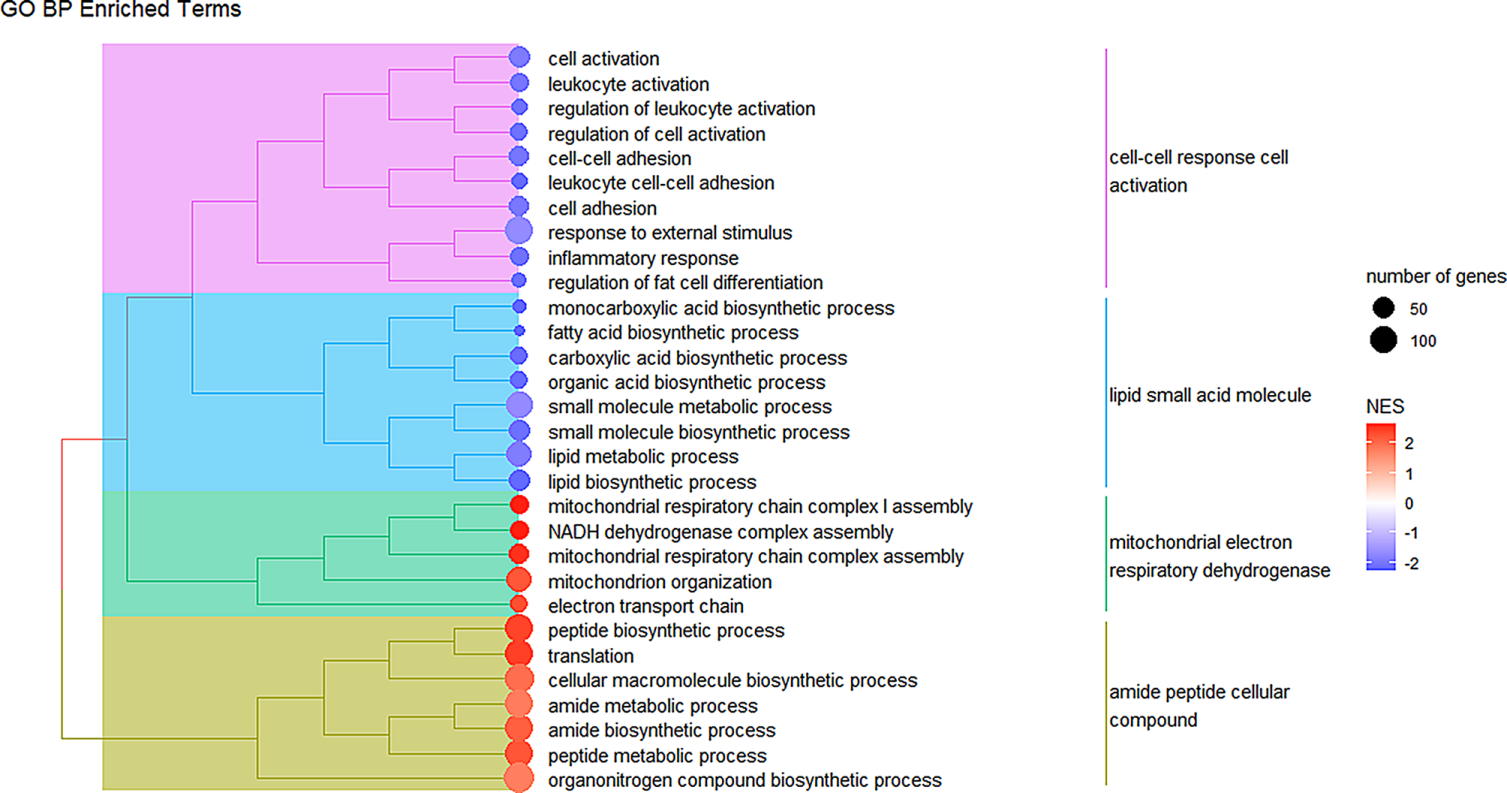
Semantic similarity treeplot of GO BP terms. A treeplot generated using the top 30 most significantly enriched BP terms. Normalized enrichment score (NES) calculated by GSEA is shown by the colored circle beside each term with positive enrichment in *QQ* marked by red and negative enrichment in *QQ* by blue. Semantic similarity analysis illustrates the broader trend of increased protein synthesis and mitochondrial activity, and decreased fat synthesis and immune activation in *QQ.*

Gene set enrichment analysis was conducted to obtain further biological insight into the changes in expression. The gene sets used for enrichment analysis were the three GO subcategories: biological process (BP), cellular component (CC), and molecular function (MF). For all GSEA analyses, terms were significantly enriched if they passed the adjusted *p*-value threshold of < 0.05. A total of 236 GO BP terms were found to be significantly enriched (Table S2, Additional File 3). These could be classified into four major groups: immune response, lipid biosynthesis and metabolism, protein biosynthesis and translation, and mitochondrial activity, with immune and lipid-related terms generally being negatively enriched in *QQ*, and protein and mitochondria-related terms being positively enriched (Fig. 2).

**Fig. 3:**
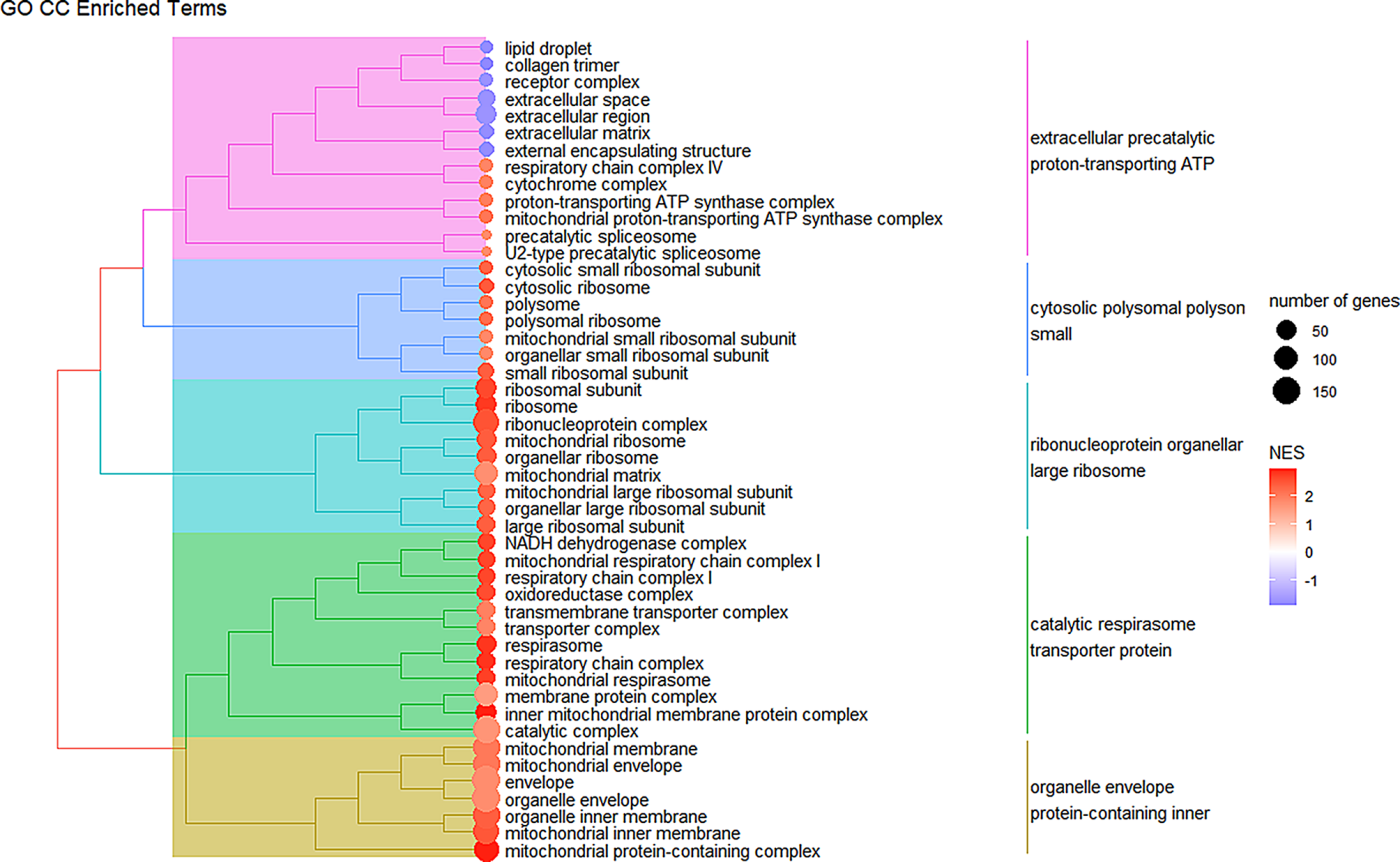
Semantic similarity treeplot of GO CC terms. A treeplot generated using all 48 of the significantly enriched CC terms. Positively enriched terms in *QQ* are denoted by a red circle, while negatively enriched terms in *QQ* are marked by a blue circle. Most cellular component terms relate to the increased ribosomal and mitochondrial transcripts in *QQ* animals.

For the GO CC terms, 48 were found to be significantly enriched (Table S3, Additional File 3). The majority of these terms are related to ribosomal and mitochondrial components, and are positively enriched in *QQ* animals, though notably a few cellular component terms such as ‘lipid droplet’ and ‘collagen trimer’ are negatively enriched (Fig. 3).

**Fig. 4:**
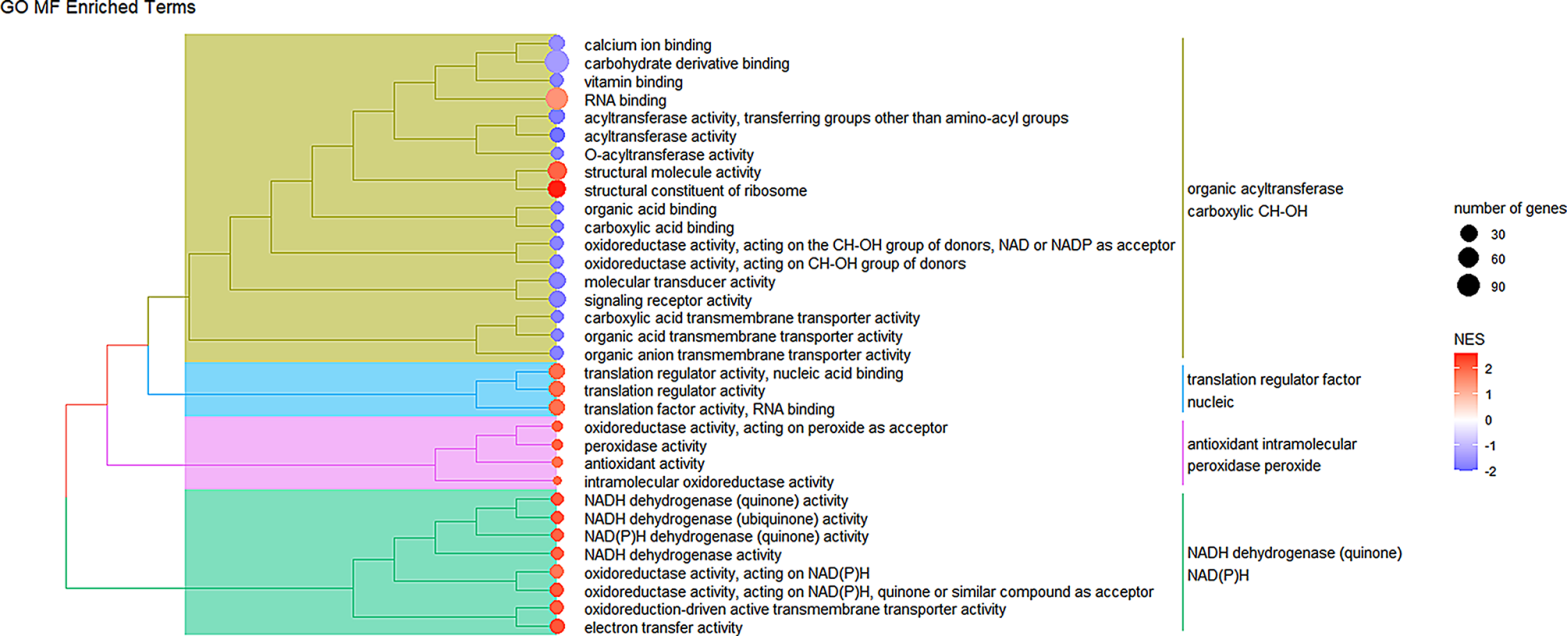
Semantic similarity treeplot of GO MF terms. **A** treeplot generated using all 33 of the significantly enriched MF terms. Positively enriched terms in *QQ* are denoted by a red circle, while negatively enriched terms in *QQ* are marked by a blue circle.

Lastly, with respect to the GO MF terms, 33 terms were identified as significantly enriched (Table S4, Additional File 3). Similarly to the other categories, terms pertaining to translation and mitochondrial activity were positively enriched in *QQ* animals (e.g. ‘translation regulator activity’ and ‘NADH dehydrogenase activity’), while terms pertaining to fat synthesis such as ‘acyltransferase activity’ were negatively enriched (Fig. 4).

Overall, the GO analysis with semantic similarity summarization demonstrated a likely increase in ribosomes and mitochondria alongside a decrease in adiposity in *QQ*. Expression of fat-related genes seems to be an important contributor to *QQ* identity and clustering in this dataset; it is likely a major contributor to why the outlier, sample 482 A, clustered with *QQ* instead of the other *qq* samples. However, it is not the sole *QQ*-defining attribute, and in other respects, the outlier followed a pattern more like the other *qq*.

### Genotype-phenotype relationship

To validate the impact of this haplotype on phenotype in the Charolais-sired population sampled from in this study, a linear mixed-effects model was constructed to test the association of haplotype with ten phenotypes: birth weight (BW), weaning weight (WW), yearling weight (YW), average daily gain (ADG), dry matter intake (DMI), hot carcass weight (HCW), ribeye area (REA), backfat (BF), kidney pelvic heart fat % (KPH), and marbling score (MS). The 344 cattle in this population were genotyped using the Illumina^®^ BovSNP50 BeadChip (50K). The variant Hapmap33628-BTC-041023 (rs110834363 / Chr6:37,505,093 T > C) was used as a surrogate for the *Q* haplotype, as it has been previously shown to be exclusive to this haplotype [58]. The results of this analysis are presented in Table S5 (Additional File 4). The *Q* haplotype was statistically significant (*p* < 0.05) for increased BW, YW, ADG, HCW, and REA, as well as decreased BF, KPH, and MS, reflecting the increased growth and decreased adiposity observed in previous studies as well as in the presented RNA-seq data.

## Discussion

The results from this RNA-seq analysis provide insight into potential mechanisms underlying the difference in growth between animals with different haplotypes for the *LCORL-NCAPG* locus or at least reflect the existing differences in the tissue of these animals at their stage of development when sampled. Since one copy of an animal’s haplotype must be passed by their sire, this does leave the possibility that other background genetics contributed by their sire may be over-represented and contribute to the differential gene expression seen in these animals, as these cattle were not derived from deliberate *Qq* x *Qq* crosses. Particularly since the *Q* haplotype is abundant in Charolais populations [8], there may be fewer sires that can contribute *q* haplotypes and thus could be potentially overrepresented in this dataset. Nevertheless, even after accounting for the effect of sire on phenotype, the *Q* haplotype still has a highly significant impact on growth and is likely significantly contributing to the differential gene expression observed in these samples.

It must be noted that using bulk RNA-seq with muscle tissue comes with the potential for significant differences in cellular composition (e.g., muscle, fat, or connective tissue) that may influence the results. For example, fat-related transcripts were highly variable between samples. However, surrogate variable correction was able to accommodate for this variance and may have been able to adjust for other unaccounted-for variance in tissue composition. The overarching trends observed in the gene set enrichment analysis suggest differences in energy partitioning between *QQ* and *qq* animals that reflect the increased lean growth observed in *QQ* animals.

### Decreased lipid synthesis & adipokine secretion in *QQ*

Most significant terms that had negative enrichment scores were related to lipid synthesis, lipid metabolism, and immune/inflammatory response. Notably, the gene with the third most-extreme log-fold change was *LEP* (LFC = -1.84; *q*-value = 0.000808), which is perhaps one of the most well-known genes related to adiposity [74]. *LEP* encodes leptin, a hormone that is responsible for mediating information on long-term energy storage between the brain and the body [75]. It is primarily synthesized by adipocytes [76], but it is known to be produced by skeletal muscle as well [77, 78]. Leptin levels are positively correlated with increased adiposity [79], and high amounts of this hormone discourage continued weight gain by suppressing appetite and promoting energy use in the body [75, 80].

Due to the fact that *LEP* is disproportionately secreted by adipocytes, it is unclear whether the difference in leptin expression between *QQ* and *qq* samples is merely reflecting the fact that *QQ* animals tend to be leaner and thus would have less fat in their muscle on average compared to *qq*, or if the difference could be due to wider downstream alterations in the transcriptome caused by a change at the *LCORL-NCAPG* locus. *LEP* expression has been found to be generally lower in leaner breeds of cattle [81]. Although decreased leptin promotes intake of food, increased leptin signals a high energy balance in the body and has been shown to promote uptake of glucose and fatty acids in skeletal muscle [82] and is even necessary at certain levels for muscle growth [83], which is somewhat contradictory with the increased growth seen in the *QQ* samples that have decreased leptin.

Other important genes involved in fat synthesis, such as fatty acid synthase (*FASN*), fatty acid elongase-5 (*ELOVL5*), ATP citrate lyase (*ACLY*), and acetyl-CoA carboxylase alpha (*ACACA*) all have significantly decreased expression in *QQ* animals. Several of these genes have been related to differences in fatty acid composition and distribution in other studies investigating gene expression in beef cattle [84–86]. Elevated expression of these genes and others in their pathway are associated with increased subcutaneous fat [86] and may be particularly elevated in periods of compensatory growth after feed restriction [84], indicating their importance for fat deposition. Interestingly, expression of *ACACA* and *FASN* has been shown to be significantly increased in Angus cattle, a breed where the *Q* haplotype is fairly rare, compared to Fleckvieh, a breed with a high frequency of the *Q* haplotype [8, 87].

As mentioned previously, immune- and inflammation-related terms were also negatively enriched alongside fat-related terms. Adipose tissue serves an endocrine role by communicating the energy storage situation of the body to the brain as well as the immune system [88]. Adipokines secreted by adipose tissue, including leptin, activate and enhance immune and inflammatory responses. This regulatory system likely exists due to the energy-intensive nature of mounting an immune response [89]. Thus, the reduced expression of these immune transcripts is most likely a direct result of the decreased adipose-associated transcripts in *QQ*.

### Increased ribosomal biogenesis & protein accretion in *QQ*

While the negative enrichment scores for lipid synthesis and immune activation correspond to the reduced fat deposition seen in *QQ* individuals, the positive enrichment of terms relating to protein synthesis and ribosome biogenesis suggest an increased capacity for protein accretion. The most statistically significant GO BP terms were the positively enriched terms relating to protein synthesis (‘amide biosynthetic process’, ‘peptide biosynthetic process’, ‘translation’, etc.), which can be accounted for predominantly by a large number of genes encoding for ribosomal proteins having significantly higher expression in *QQ* animals.

Postnatal growth of skeletal muscle tissue is largely due to hypertrophy of the muscle fibers, rather than the creation of new fibers [90]. This hypertrophy occurs by the accretion of more protein within the individual fiber and is ultimately limited by the rate the cell can synthesize more protein. This rate is dictated by the muscle fiber’s ability to create sufficient transcripts, which is determined by its number of nuclei and can be increased by the proliferation and incorporation of satellite cells [90], and by its ability to translate those transcripts into protein, which is limited by the cell’s quantity of ribosomes [91, 92]. Thus, this increased expression of ribosomal protein transcripts in *QQ* animals could indicate an increase in ribosomal biogenesis, and therefore more capacity for muscle hypertrophy and lean growth. As *QQ* animals are known to have increased average daily gain [93], this greater ribosomal capacity may partly explain their ability to grow at a faster pace.

In addition to ribosomal proteins, eukaryotic initiation factors *EIF2D* and *EIF3K* also had increased expression in *QQ* individuals. In mammals and other eukaryotes, initiation of translation is often the rate-limiting step of protein synthesis, and is dictated by eukaryotic initiation factors [94]. Thus, the upregulation of these initiation factors would be required in cells whose protein synthesizing capacity was increased for that capacity to be put to productive use. Eukaryotic initiation factor 3 subunit K (EIF3K) is an optional subunit of EIF3. Duan et al. [95] demonstrated that depletion of EIF3K does not actually decrease translation, but instead decreases the selectivity of translation. In other words, EIF3K acts to regulate the available translational capacity of the cell. Curiously, that same study found that cells depleted of EIF3K actually had an increase of ribosomal protein transcription and translation, which is the opposite of what is occurring in *QQ*. Eukaryotic initiation factor 2D (EIF2D) on the other hand, seems to be most involved in ribosomal recycling and translation re-initiation, maximizing the ‘uptime’ of ribosomes present in the cell by keeping them available for new tasks [94]. The fact that these two eukaryotic initiation factors are oriented toward making efficient use of available ribosomes and that ribosomal protein transcripts are also expressed more suggests that there may be an increased demand for protein synthesis in *QQ* individuals, perhaps promoting the increased lean growth in these animals.

### Changes to mitochondrial activity in *QQ*

Among the enriched cellular component GO terms, 24 out of 48 were related to mitochondrial function, with all being positively enriched in *QQ* animals. This, alongside enrichment of several mitochondria-related terms in the BP and MF ontologies (‘electron transport chain’, ‘mitochondrial respiratory chain complex I assembly’, ‘oxidoreduction-driven active transmembrane transporter activity’, etc.) implies an increased quantity of mitochondria present in *QQ* samples, or perhaps an increase in biogenesis of mitochondria in *QQ*. Most of the mitochondria-related transcripts that have increased expression in *QQ* originate from the nuclear genome and consist of genes encoding for components of complex I (*NDUFA7*,* NDUFA13*, *NDUFC1*, *NDUFB1*, etc.), ATP synthase membrane subunits (*ATP5MF*, *ATP5ME*, *ATP5MJ*), and membrane translocases (*TIMM8B*, *TOMM7*, *TOMM6*), among others.

Paradoxically, several transcripts directly encoded by the mitochondrial genome, such as *ND5*, *ND4L*, *ND6*, *ND1*, and *ATP8*, are among the most significantly associated with the *QQ* haplotype yet have decreased expression in *QQ*. One possible explanation for this is that there may be fewer mitochondria present in *QQ* animals and thus, fewer mitochondrial chromosomes from which these transcripts could be synthesized. In this scenario, the positively enriched mitochondrial terms would suggest increased biogenesis of mitochondria in *QQ*, perhaps as a consequence of the reduced mitochondria present, though it then is unclear why there is an increase in expression of nuclear transcripts contributing to complex I formation, but not a corresponding increase in mitochondrial transcripts.

Taken at face value, the aforementioned positive enrichment of GO BP and MF terms suggest increased mitochondrial activity. The positive enrichment of oxidoreductase acting on NAD(P)H, alongside the negative enrichment of oxidoreductase with NAD(P) + as the acceptor, indicate a shift in the cellular metabolism in *QQ* animals. As processes where NAD(P)H acts as the reducer are generally anabolic [96] and processes where NAD(P) + is the acceptor tend to be catabolic [97], it would appear that animals with the *QQ* haplotype are in an anabolic state, which would agree with their increased growth phenotype. It is worth noting that both increased lipid synthesis and increased protein synthesis can demand increased mitochondrial activity, as these anabolic processes are energy intensive. The transcriptional differences observed in this study would suggest both decreased lipid synthesis and increased protein synthesis; as these have opposed effects on the energy demand of the cell, this could mean that the energy demands for increased protein synthesis outweigh the decrease in fat deposition occurring in *QQ* animals. This could also be connected to the contradictory effects seen in the expression of mitochondrial genes; however, more rigorous work would need to interrogate the potential differences in energy partitioning that may exist between animals of different *LCORL-NCAPG* haplotypes.

Among the autosomal transcripts encoding for mitochondrial components that had significantly increased expression, coiled-coil-helix-coiled-coil-helix domain containing 7 (*CHCHD7*) was present. The protein product encoded by *CHCHD7* is thought to work in the intermembrane space of the mitochondria, but its exact function remains unknown [98]. *CHCHD7* appears in networks connected to *LCORL* and *NCAPG*, however, this connection is derived by textmining, and is likely due to *CHCHD7* being a part of the *PLAG1* locus, another region of the genome known for its influence on stature [99]. *CHCHD7* and *PLAG1* are 500 bp apart in the bovine genome and share a promoter region. A study investigating the *PLAG1-CHCHD7* locus in cattle observed increased expression of *CHCHD7* and *PLAG1* being associated with the locus, and identified a variant in their shared promoter region that could be causative [100]. Unfortunately, *PLAG1* did not pass the logCPM filter required to be considered for the differential gene expression analysis in the present study, so we were unable to discern if expression of *PLAG1* was increased alongside *CHCHD7*. However, it is possible that the transcriptional changes caused by the *LCORL-NCAPG* locus could be affecting the transcription of other genes associated with growth, such as these.

### Important DEGs underlying growth pathways

Several genes that were differentially expressed between *QQ* and *qq* animals have been documented as having important roles for growth but did not necessarily fit into the previously discussed overarching gene ontology categories. For example, *GHR* (growth hormone receptor) is among the most significant differentially expressed genes, with reduced expression in *QQ* animals. *GHR* dictates the responsiveness of cells to growth hormone, also known as somatotropin. Growth hormone is important for skeletal and muscle growth [101], and mice with *GHR* knocked-out exhibit dwarfism while also tending toward obesity [102]. In humans, *GHR* tends to have higher expression in leaner individuals [103], which conflicts with the leaner phenotype associated with *QQ*. However, *GHR* is also known to have biased expression in fat cells compared to skeletal muscle [104], so the difference in *GHR* expression may again be a manifestation of the difference in adipose tissue present between *QQ* and *qq* individuals.

Similarly, *FGF1* (fibroblast growth factor 1) also has significantly lower expression in *QQ* individuals. While this gene is perhaps most known for its important role in stimulating cellular division and embryonic development [105], it is also highly expressed in adipocytes, particularly after differentiation [106]. Thus, the decreased expression of *FGF1* may also be another indicator of reduced adiposity in *QQ* animals.

Interestingly, insulin-like growth factor 2 (*IGF2*) and insulin-like growth factor 2 binding protein 2 (*IGF2BP2*) have significantly higher expression in *QQ* animals, while expression of *IGFBP4* (insulin-like growth factor binding protein 4) is significantly reduced. Though *IGF2* was believed to be involved predominantly in prenatal growth [107], it has been observed to be postnatally expressed in pigs and cattle [108, 109]. In fact, recent research suggests that *IGF2* expression is not restricted to prenatal and neonatal periods and *IGF2* actually has considerably high postnatal expression in many species [110]. Increased postnatal expression of *IGF2* has been shown to be positively associated with growth in pigs and mice [109, 111], and given the well-established evidence of IGF1 and IGF2 in promotion of growth [107, 112], it certainly is possible that *IGF2* could be mediating the increased growth associated with the *Q* haplotype.

IGF1 and IGF2 both mediate their effects and are regulated by specific binding proteins that are divided into two classes, IGFBP and IGF2BP. The distinction between these classes is based on whether the protein binds to the growth factor peptide or transcript; IGFBPs bind to the secreted IGF1 and IGF2 peptides [112], while IGF2BPs bind to the mRNA of *IGF2* and *IGF1R* and regulate their translation [113]. As previously mentioned, two IGF binding proteins were found to be differentially expressed. IGFBP4 acts as a traditional binding protein and is typically inhibitor of IGF activity, though mice with IGFBP4 knocked out actually have decreased growth [114]. Given the inhibitory activity of IGFBP4, its slight downregulation in *QQ* (-0.33078 log-fold change, *q-*value 0.019153) suggests a potential increase in IGF activity. In the case of IGF2BP2, this protein binds to the 5’ UTR of the *IGF2* mRNA, and promotes its translation when activated by mTOR complex 1 [113, 115], integrating translation of *IGF2* with various other nutrient and energy level signals [116]. Since *IGF2BP2* expression is increased in *QQ* animals, this implies that *IGF2* is not just more highly expressed in these individuals but may be translated and secreted at a higher rate as well.

Given that the difference between *QQ* and *qq* animals in this study comes down to the presence or absence of a selected-upon haplotype encompassing *LCORL* and *NCAPG*, it is naturally of interest to consider if either of these genes are differentially expressed, as the difference in expression of either of these genes could potentially be responsible for the up- or down-regulation of the other DEGs, either directly or indirectly. In these data, *NCAPG* was not expressed highly enough to pass the filtering step (mean CPM 1.09; only 5 samples passed the 1.5 CPM filter threshold, three of which were *QQ* and two were *qq*). However, *LCORL* was among the genes expressed significantly higher in *QQ* animals (*q*-value = 4.102E-05), with a log-fold change of 0.5633147, or 1.477660-times greater expression in *QQ* compared to *qq*. While *LCORL* is likely able to mediate changes in transcription in its short isoform as a transcription factor [117] or its long isoform by accessorizing with PRC2 [52], the present lack of functional data makes it difficult to discern which, if any, differentially expressed genes found within the current study could be directly attributed to changes in *LCORL* expression.

The resolution of this study unfortunately did not permit investigation into expression of specific isoforms of *LCORL*, particularly due to the low expression of the long isoform of *LCORL* in muscle tissue (data not shown). One of the variants defining the *Q* haplotype appears to result in the truncation and possible loss of function of the long isoform of *LCORL* [58]. As this isoform encodes for an accessory subunit of PRC2, which is known for its essential role in formation and maintenance of cellular identity via H3K27me3 [54], it is possible the loss of function of this isoform may be responsible for some of these broad transcriptional changes, directly or indirectly. In the case of *IGF2* in particular, a recent publication found that a region of H3K27me3 nearby *IGF2*, that they referred to as an “*IGF2* looping silencer,” was strongly impactful on *IGF2* expression [118]. This evidence further supports the possible connection between this loss of function mutation of *LCORL*, its impact on PRC2, *IGF2* expression, and growth.

## Conclusions

This RNA-seq analysis found significant differential gene expression between muscle tissue samples between *QQ* and *qq* animals reflective of broader changes in tissue composition and perhaps molecular-level differences that may be driving their phenotype. Generally, the trend of increased expression of ribosomal and mitochondrial transcripts and the reduced expression of lipid transcripts implies a shift toward protein accretion and away from fat deposition. While the upregulation of *LCORL* and *IGF2* provides a compelling implication of a network underlying the increased growth and transcriptional changes, much work needs to be done to follow up on the hypotheses suggested by these findings. It would be of particular value to validate whether there is an increased number of ribosomes and/or mitochondria in the muscle of animals with the *QQ* haplotype, or how transcription between *QQ* and *qq* differs when comparing cells of the same type versus bulk RNA-seq to potentially better control for confounded tissue composition differences. Further exploration of which genes may or may not be direct targets of either of the isoforms of *LCORL* or affected indirectly by downstream transcriptional changes would also be useful. Nevertheless, our findings provide further evidence that the *Q* haplotype of *LCORL-NCAPG* locus is associated with substantial changes in the transcriptome that may be underlying the difference in growth.

## Electronic supplementary material

Below is the link to the electronic supplementary material.

Supplementary Material 1

Supplementary Material 2

Supplementary Material 3

Supplementary Material 4

## Data Availability

The raw RNA-seq reads and normalized counts generated in this study is available in the Gene Expression Omnibus (GEO) repository, accession number GSE98736. https://www.ncbi.nlm.nih.gov/geo/query/acc.cgi?acc=GSE98736.

## Abbreviations

QTL: Quantitative trait locus / loci
QTN: Quantitative trait nucleotide
BTA6: Bovine chromosome 6
LD: Linkage disequilibrium
PRC2: Polycomb repressive complex 2
FDR: False discovery rate
GSEA: Gene set enrichment analysis
GO: Gene ontology
BP: Biological processes
CC: Cellular component
MF: Molecular function
BW: Birth weight
WW: Weaning weight
YW: Yearling weight
ADG: Average daily gain
DMI: Dry matter intake
HCW: Hot carcass weight
REA: Ribeye area
BF: Backfat
KPH: Kidney pelvic heart fat %
MS: Marbling score
