# Supplementary figures and images for: Characterizing differences in the muscle transcriptome between cattle with alternative LCORL-NCAPG haplotypes
Source: BMC Genomics. 2025 May 14;26:479. doi: 10.1186/s12864-025-11665-z (PMC12076881; doi:10.1186/s12864-025-11665-z)

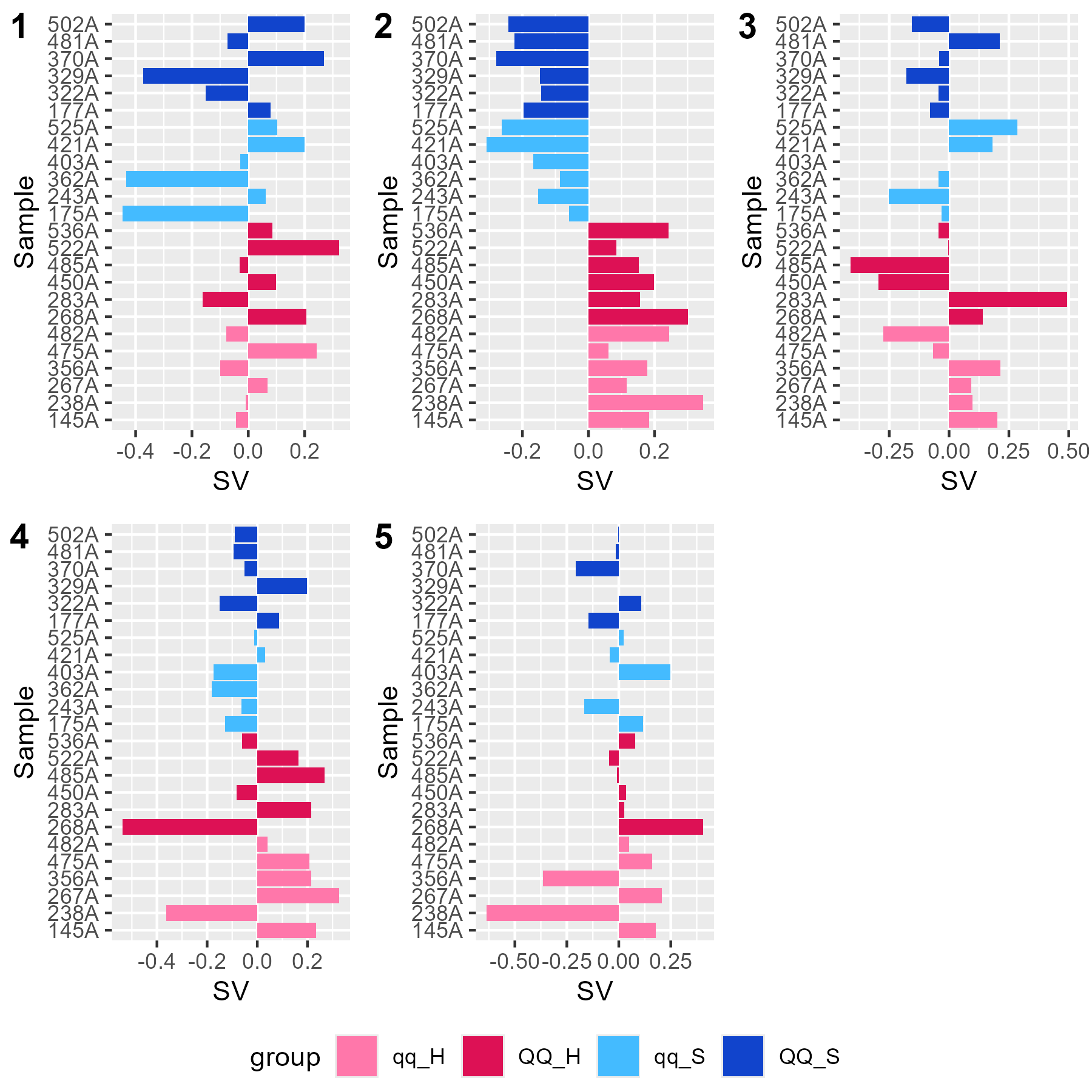

Supplement: Supplementary file 1 — Supplementary Material 1 [file 12864_2025_11665_MOESM1_ESM.png]

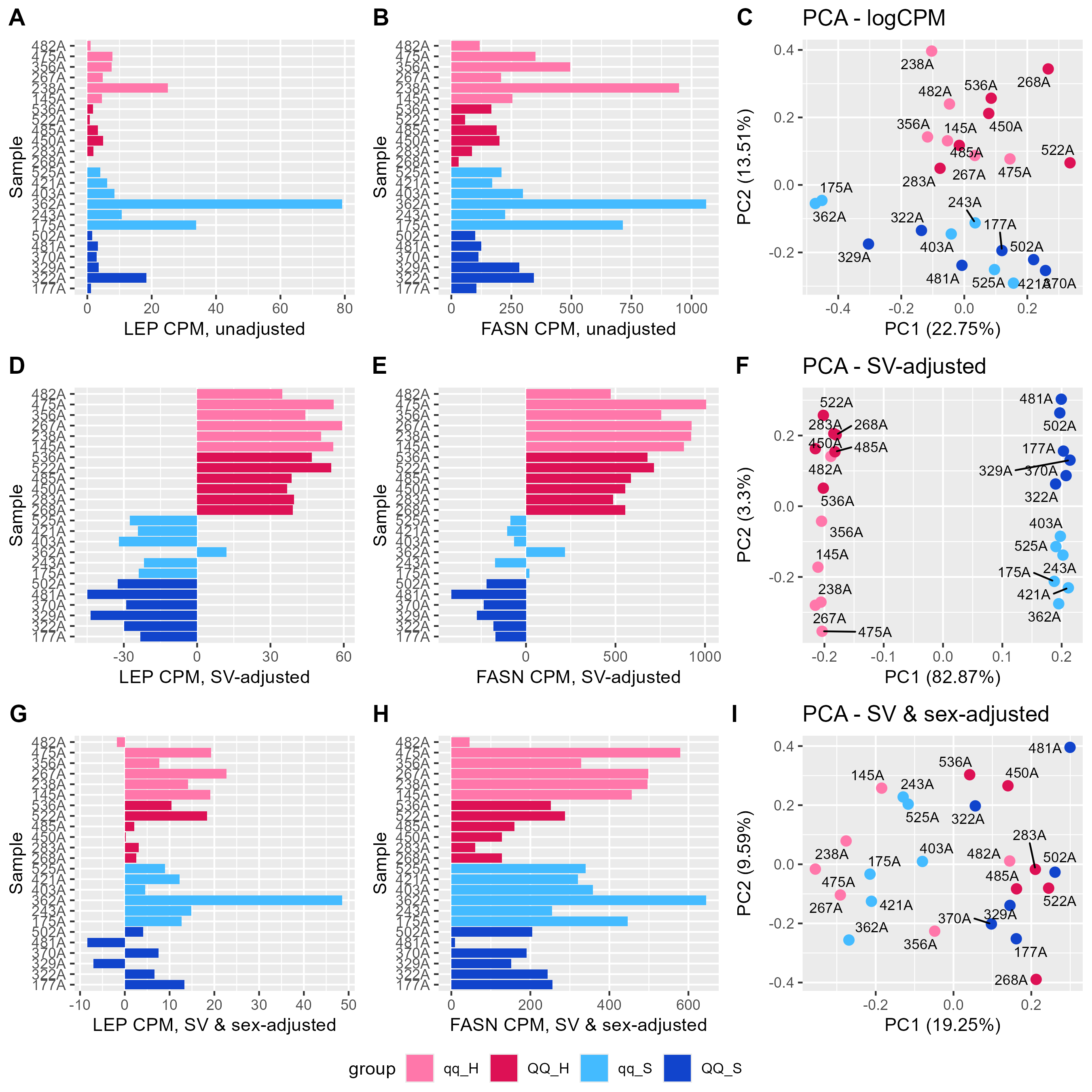

Supplement: Supplementary file 2 — Supplementary Material 2 [file 12864_2025_11665_MOESM2_ESM.png]
